# Towards assessing cortical bone porosity using low-frequency quantitative acoustics: A phantom-based study

**DOI:** 10.1371/journal.pone.0182617

**Published:** 2017-09-07

**Authors:** Florian Vogl, Benjamin Bernet, Daniele Bolognesi, William R. Taylor

**Affiliations:** Institute for Biomechanics, ETH Zurich, Zurich, Switzerland; University of Memphis, UNITED STATES

## Abstract

**Purpose:**

Cortical porosity is a key characteristic governing the structural properties and mechanical behaviour of bone, and its quantification is therefore critical for understanding and monitoring the development of various bone pathologies such as osteoporosis. Axial transmission quantitative acoustics has shown to be a promising technique for assessing bone health in a fast, non-invasive, and radiation-free manner. One major hurdle in bringing this approach to clinical application is the entanglement of the effects of individual characteristics (e.g. geometry, porosity, anisotropy etc.) on the measured wave propagation. In order to address this entanglement problem, we therefore propose a systematic bottom-up approach, in which only one bone property is varied, before addressing interaction effects. This work therefore investigated the sensitivity of low-frequency quantitative acoustics to changes in porosity as well as individual pore characteristics using specifically designed cortical bone phantoms.

**Materials and methods:**

14 bone phantoms were designed with varying pore size, axial-, and radial pore number, resulting in porosities (bone volume fraction) between 0% and 15%, similar to porosity values found in human cortical bone. All phantoms were manufactured using laser sintering, measured using axial-transmission acoustics and analysed using a full-wave approach. Experimental results were compared to theoretical predictions based on a modified Timoshenko theory.

**Results:**

A clear dependence of phase velocity on frequency and porosity produced by increasing pore size or radial pore number was demonstrated, with the velocity decreasing by between 2–5 m/s per percent of additional porosity, which corresponds to -0.5% to -1.0% of wave speed. While the change in phase velocity due to axial pore number was consistent with the results due to pore size and radial pore number, the relative uncertainties for the estimates were too high to draw any conclusions for this parameter.

**Conclusions:**

This work has shown the capability of low-frequency quantitative acoustics to reflect changes in porosity and individual pore characteristics and demonstrated that additive manufacturing is an appropriate method that allows the influence of individual bone properties on the wave propagation to be systematically assessed. The results of this work opens perspectives for the efficient development of a multi-frequency, multi-mode approach to screen, diagnose, and monitor bone pathologies in individuals.

## Introduction

Osteoporosis is a systemic bone pathology characterized by the degradation of micro- and macroscopic bone properties [1–3] and a corresponding increase in fracture risk. Osteoporosis is the most widespread skeletal disorder, affecting one in two women and one in five men over the age of 50 in the western world [4,5] and more than 200 million individuals worldwide [2]. The lifetime risk of suffering from an osteoporosis-related fracture in subjects over 50 years is estimated to be 53.2% in women and 20.7% in men [6], a number that will likely be exacerbated by increasing life expectancies. Apart from causing individual suffering, osteoporosis also places a high financial burden on the public health sector, estimated to be $3.8 billion annually in the US alone [1,6], with every osteoporosis-induced fracture incurring some $10’000 additional cost within the initial six months after fracture [7].

Although considerable changes occur within the trabecular bone structure due to osteoporosis [5,8], the endosteal cortex is also known to be affected, where an imbalance in bone resorption over bone formation leads to an increase in cortical porosity, as well as thinning of the cortical shell, thereby increasing bone fragility [5,9–12]. Cortical porosity has also been shown to account for most of the variations in mesoscopic bone elasticity and anisotropy [13], making cortical porosity a key property governing the mechanical properties of the bone. Thus, any state-of-the-art approach to clinically assess bone health clearly needs to take cortical porosity into account.

Axial transmission quantitative acoustics (ax-QA)—here assumed to include quantitative sound in the frequency range between 1Hz and 20kHz as well as quantitative ultrasound when frequencies over 20k Hz are employed—is a bone sonometry method that has shown sensitivity to bone related pathologies [14–19] and individual properties of human cortical bone [20–23]. Compared to conventional radiation-based methods, ax-QA is fast, non-invasive, portable, inexpensive, and radiation-free, which opens perspectives for applications such as diagnosis, monitoring, and widespread prevention programs. In ax-QA, transducers are placed supercutaneously along the bone (e.g. tibia, ulna…) and transmit acoustic waves into the bone cortex through the overlying soft-tissues. Separate surface sensors measure the propagation of these acoustic waves, providing information on bone properties such as cortical thickness [20,21] or porosity [22,23].

Early ax-QA devices used acoustic waves of frequencies between 250kHz and 1MHz and analysed the “first arriving signal” (FAS), sometimes also termed the “speed of sound” or “fast wave”, which has commonly been defined by the first crossing of the signal over an arbitrary threshold and has therefore never been standardized [15,21,24–28]. Later, this FAS approach was extended to also consider the “energetic late arrival” (ELA) or “slow wave”, which is a high-energy signal arriving after the FAS. It was eventually found that the FAS and ELA consisted of not only one, but rather multiple wave modes and their reflections propagating within the bone at the same time, making valid interpretation of results difficult [29,30]. Specifically, it has been shown through numerical and experimental studies that the FAS corresponds to a lateral head wave for λ << d and to a S0-like guided wave (lowest-order symmetric) from plate theory for λ > d; the energetic-late-arrival (ELA) was identified to correspond to a Rayleigh wave for λ << d and to the A0-like guided wave (lowest-order antisymmetric) for λ > d [30–32], where λ denotes the wavelength and d the cortical thickness.

Furthermore, it was shown that due to their intrinsic characteristics, particular wave modes are more sensitive to the measurement of specific bone properties than others and that these characteristics can vary drastically with frequency [5,16,21,30,33]. Together, these insights have led to the development of multi-mode analyses as well as full wave analyses of individual modes [31,34], in which the complete time-signal is considered. Contrary to FAS and ELA, which only assess a small part of the signal, such full wave analyses allow the differentiation between various reflections propagating within the bone without the requirement for arbitrary thresholds.

The fact that the ability to measure a certain bone property in a sensitive manner depends on the frequency and wave mode opens perspectives for a multi-frequency, multi-mode analysis to gather comprehensive information on bone properties over various length scales and various depths of the bone [23,33]. Towards bringing such an approach to clinical application, considerable effort has been invested in assessing the potential information accessible over different frequency ranges through in-vitro [21,29,31,34] as well as in-vivo studies [16,19]. By examining the A0- and S0-modes in the frequency range from 250 kHz to 1.25 MHz, these experiments have clearly shown that information on geometrical properties and properties near the endosteum are only accessible using frequencies below about 500 kHz in the case of these modes, but not by using higher-frequency waves due to their limited penetration depth into the bone [23,30,31,35]. The quantification of geometrical and endosteal properties is critical, particularly in the assessment of osteoporosis, in which the changes in bone properties such as porosity mainly occur at the endosteal surface [10,11]. While recent research suggests that these properties might still be accessible at frequencies above 500kHz using higher order modes [36–38], such approaches are still restricted by the complexity of exciting, measuring, and differentiating the individual modes. While these higher order modes have the potential to be sensitive to additional characteristics of bone properties over and above the standard low-order modes, the use of these A0- and S0-modes offers a robust and established way to assess geometry and endosteal properties at a macroscopic level.

In addition to the problem of finding suitable frequency ranges and wave modes, experiments on in-vitro or in-vivo human bone are complicated by an entanglement of the effects of individual characteristics (e.g. geometry, porosity, anisotropy, overlying soft-tissue) on the measured wave propagation. In order to address this entanglement problem, we therefore propose a systematic bottom-up approach, in which only one bone property is varied while all others are kept constant, before addressing the various interaction effects.

The aim of this study was therefore to investigate the capability of low-frequency ax-QA using the fundamental A0-mode to measure porosity in bone phantoms in a systematic manner, examining the role of varying pore size, as well as axial and radial pore number, while keeping geometry and material properties constant.

## Methods

### Bone phantoms

**Fig 1:**
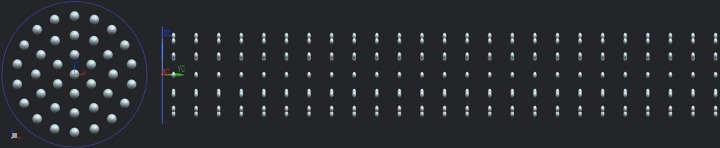
In this study phantoms with varying porosities in radial (left) and axial (right) direction were manufactured. The radial distribution is identified by the number of pores in the cross-section (here 37), and the axial distribution is identified by the number of such patterns along the axis (here 25).

For this study, 14 bone phantoms with varying pore size, radial or axial pore number were designed (see Fig 1 and Table 1) to allow the investigation of porosities (volume fraction) between 0% and 15%. These values were chosen in order to mimic human bone, which has a cortical porosity between approximately 1% and 20%, depending on age, bone, and osteoporotic status [12,39–43].

**Table 1:** Design parameters of the manufactured phantoms.

id	porosity (%)	pore size (mm)	pore pattern	# of patterns	# of pores
100	0.00	0	0	0	0
101	0.12	0.5	19	25	475
102	0.94	1	19	25	475
103	3.17	1.5	19	25	475
104	7.51	2	19	25	475
105	14.66	2.5	19	25	475
107	1.84	1	19	49	931
108	2.74	1	19	73	1387
109	3.64	1	19	97	1843
110	1.01	1	7	73	511
111	1.87	1	13	73	949
113	3.60	1	25	73	1825
114	5.34	1	37	73	2701

The column “id” contains an arbitrarily chosen identifier for each phantom, “pore pattern” refers to the number of spheres in one cross-sectional pattern, “# of patterns” refers to the number of such cross-sectional patterns, and “# of pores” gives the overall number of pores.

All phantoms were cylindrical with 3cm diameter and 30cm length, and were manufactured out of Polyamid 12 (PA12) using selective laser sintering in a vertical orientation. The simple phantom shape approximates a long bone in the human body (e.g. tibia, ulna, femur), avoids the effects of more complex geometry or wall thickness on the wave propagation, and facilitates an experimental procedure with high reproducibility and repeatability. To check whether the laser sintering manufacturing process or the manufacturing orientation introduces additional anisotropic effects on the wave propagation, one additional phantom was manufactured in a horizontal orientation and two further phantoms of the same shape, made from Polyvinylchlorid and Polyethylen, were manufactured by conventional extrusion. Additionally, planar X-ray images were taken with a fluoroscope (resolution 0.27 mm) to qualitatively confirm the success of the manufacturing process with regard to the design specifications.

### Measurement setup and protocol

All acoustic measurements in this study were performed using the Bone Stiffness Measurement Device (BSMD), which has been described in detail elsewhere [44] but a brief overview is presented here: The BSMD consists of a piezo-electric transducer to excite an acoustic wave, multiple acceleration sensors to measure the wave propagation, and a data acquisition system to control the device and to record the measurement data. The acoustic wave was excited by a sine of 3300 Hz, enveloped by a Gaussian with Full-Width-at-Half-Maximum (FWHM) of 2000 Hz. The accelerometers (4518, Brüel & Kjael GmbH, Pöcking, Germany) had a sensitivity of 100 mV/G, where G is 9.81 m/s^2^, and data was sampled at a frequency of 96kHz.

This experiment utilised a custom-made mounting platform to achieve high reproducibility in transducer and specimen placement while also ensuring acoustic decoupling. A foam underground for the specimen provided mechanical stability for the specimens as well as a strong impedance mismatch with the specimen material. This mismatch causes near perfect reflections to occur at the phantom-foam interface and thus minimizes the effect of the foam on the wave propagation.

The transducer was mounted, facing radially inwards, 5mm from the end of the phantom, which also acted as the origin of the coordinate system. This placement allowed the excitation of a transversal wave inside the phantom, superimposing the direct wave and the first reflection from the nearest end of the phantom, thus creating a well-defined wave front propagating away from the origin. One acceleration sensor was placed in-line with the transducer at a distance of 50mm. In this configuration, a wave was induced and 2000 data points were measured, corresponding to approximately 20ms. The sensor was then repositioned 10mm along the phantom’s axis and another measurement was performed; this process was repeated until the sensor reached the end of the phantom. Overall, this procedure resulted in a collection of 26*2000 space-time data per phantom.

To exclude the possibility that the pore patterns cause an angular dependency of our results, two of the phantoms were tested in orientations between 0° and 180° in 15° steps.

### Wave speed analysis

Each of the measurements was first windowed along the time-axis using a Hamming-window of 1000 points width and center at 0.01s, followed by a 2D-Fast Fourier Transform (2D-FFT) with lengths of 256 and 32768 points, which were chosen to be about 10 times the length of acquired data for space and time respectively. This procedure transformed the space-time data, collected for each phantom, to the wavenumber-frequency domain and allows differentiation between the various possible modes at each frequency. Since the comparatively short length of the phantoms, together with the excitation duration and the wave velocity, lead to multiple reflections propagating and overlapping within the phantom, the presented 2D-FFT technique is especially advantageous for differentiating these various reflections.

A sinc function fit

$$y(x)=A\frac{sin(\pi (x-x_{0}))}{\pi (x-x_{0})}$$ (1)

was performed between 3.0 and 10.5 1/m for each frequency, corresponding to wavespeeds of approximately 270 m/s to 1200 m/s for the considered frequency range, by minimizing a sum of squared deviations cost function, where *x* is the inverse wavelength, and *x*_0_ and *A* are free parameters to be determined. This fitting function allows the isolation of the A0-like flexural wave mode travelling away from the transducer and the fitting function was motivated by the fact that the finite measurements in space can be seen as a subset of an infinite number of measurements that are contained within a rectangular window, the transformation of which is the sinc-function.

**Fig 2:**
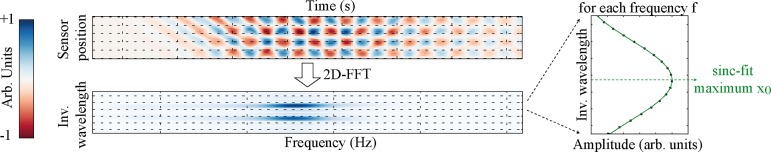
Illustration of the analysis procedure.

Using the *x*_0_ determined from the fit, the wave speed was then calculated as

$$c=c(f)=f\;\lambda (f)∼f/x_{0}(f),$$ (2)

where f is the frequency and *λ* is the wavelength. Fig 2 shows an illustration of the overall analysis procedure up this point.

The uncertainty in the wave speed Δ*c*_*fit*_, attributable to the fitting procedure, was calculated from Eq 2 using Gaussian error propagation [45]:

$$\Delta c_{fit}=\sqrt{{\left(\frac{\partial c}{\partial x_{0}}\right)}^{2}\Delta x_{0}^{2}}=\sqrt{{\left(\frac{f}{x_{0}}\Delta x_{0}\right)}^{2}},$$ (3)

where Δ*x*_0_ is the uncertainty in *x*_0_ estimated from the fitting procedure [46–48]. Finally, the determined wave speeds c(f) were averaged over a number of 100Hz frequency bands to reduce effects from the limited frequency resolution–based on our central frequency and bandwidth, we used 2900-3000Hz, 3100-3200Hz, 3300-3400Hz, and 3500-3600Hz.

### Statistics and analytical model

To estimate the overall uncertainty for each phase velocity measurement, the experiment and analysis were repeated seven times for a single phantom with full repositioning of the phantom, transducer, and sensors after each measurement. The standard deviation of the phase velocity results was then bias-corrected and used as an estimate for the overall uncertainty.

To assess the dependency of the phase velocity on porosity, weighted linear regressions were calculated for each of the pore characteristics under investigation (pore size, radial pore number, axial pore number), using the inverses of the estimated overall uncertainties as the weights.

Timoshenko beam theory was used to model the wave propagation in the bone phantoms [49]: the dispersion equation for harmonic waves was then given by

$$\frac{E\;I}{\rho A}\;\gamma^{4}-\frac{I}{A}\left(1+\frac{E}{G\;\kappa }\right)\gamma^{4}c^{2}-\gamma^{2}c^{2}+\frac{\rho I}{G\;A\;\kappa }\gamma^{4}c^{4}=0,$$ (4)

where E is the elastic modulus, G is the shear modulus, I is the area-moment of inertia for the cross-section, A is the cross-sectional area, *ρ* is the density, c is the phase velocity, *κ* is the Timoshenko shear coefficient (0.847 for this study), and *γ* = 2π/λ with *λ* being the wavelength. From Eq 4 the phase velocity c was calculated as a function of frequency using the material constants supplied by the manufacturer: *ρ* = 0.970 *g*/*cm*^3^, E = 1500 MPa, G = 532 MPa, and the geometry of the phantoms.

## Results

No differences in wave propagation were found either due to manufacturing direction or due to the angular testing orientation of the phantom. The laser sintered phantoms without porosity and the extruded phantoms all exhibited wave propagation consistent with the values predicted by Timoshenko-beam theory for the respective materials [49]: Timoshenko wave theory predicted a wave velocity value of 397 m/s (at 3kHz) and 430 m/s (at 3.6 kHz) for the laser-sintered phantoms without porosity, corresponding to wavelengths of 13.2 cm (at 3kHz) and 11.9 cm (at 3.6 kHz). These predictions agreed with the experimentally found increase of the 0^th^ order fitting parameter c0 with frequency to within 10% of the absolute values.

The overall uncertainties of the phase velocities were estimated as 10.3 m/s, 7.6m/s, 6.7m/s, and 6.4 m/s, for the frequency bands 2900-3000Hz, 3100-3200Hz, 3300-3400Hz, and 3500-3600Hz respectively. The contribution of the uncertainties attributable to the fitting procedure derived from Eq 3 were about 5% of the overall uncertainty and thus considered negligible with respect to other error sources.

**Fig 3:**
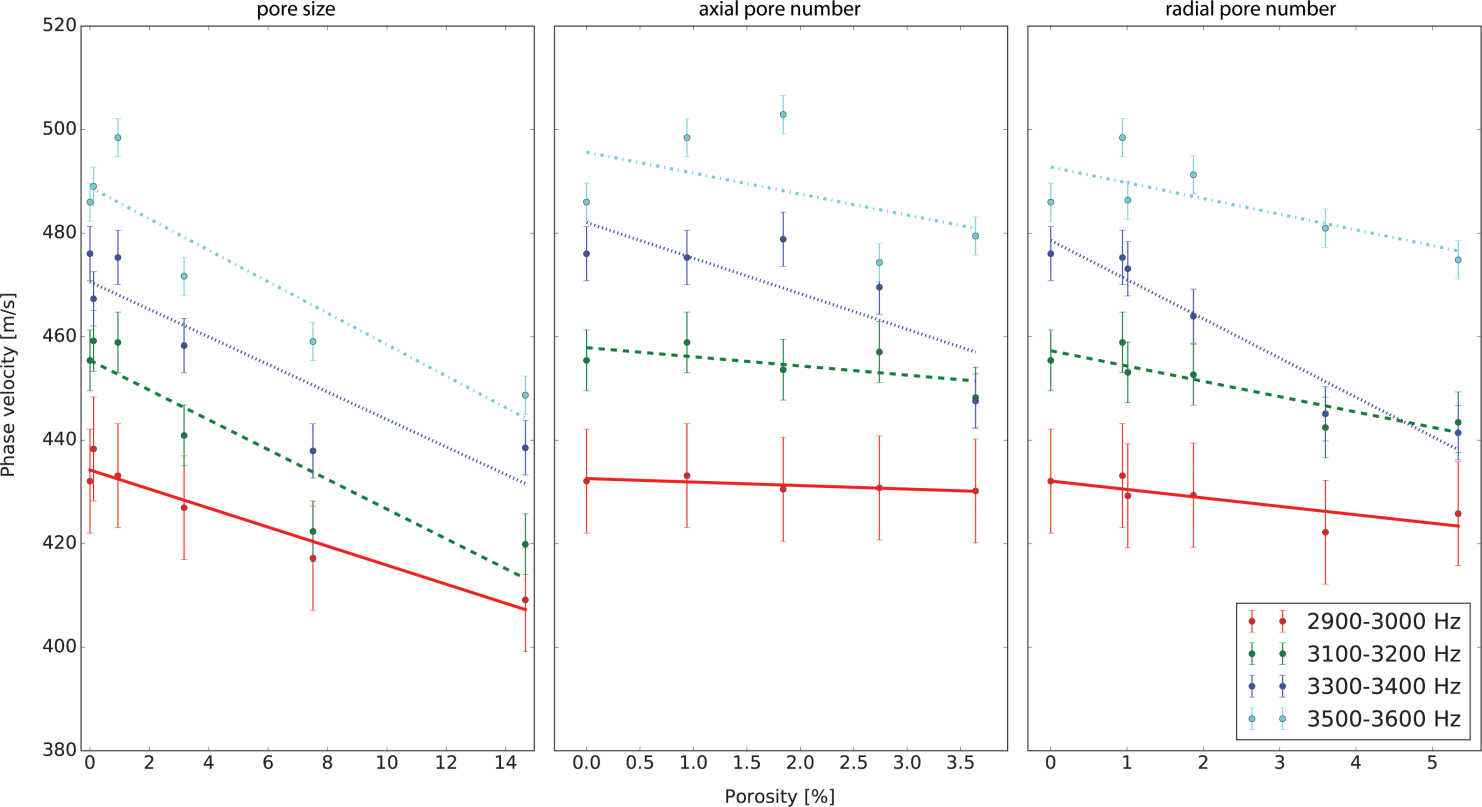
Phase velocity as a function of porosity (pore volume fraction), caused by an increase in pore size (left), radial pore number (middle), or axial pore number (right). Note the varying abscissae.

Wave speed exhibited a clear dependency on porosity, as caused by a variation in pore size, radial pore number, and axial pore number (Fig 3): For increasing pore size the wave velocity decreased by 2.47–3.40 m/s per percent of porosity; for increasing radial pore number the wave velocity decreased by -2.87–4.49 m/s per percent of porosity; and for increasing axial pore number the corresponding p-values were all greater than 0.1, which we did not consider statistically significant (Table 2).

**Table 2:** Optimized parameters, standard errors (SE), and statistics for the phase velocity as determined by weighted linear regression c = c1 · porosity + c0.

Frequency	c0	SE c0	c1	SE c1	R^2^	p-value
**Size**						
(2900, 3000)	437.19	3.44	-2.47	0.50	0.858	<0.01
(3100, 3200)	454.04	4.23	-2.79	0.61	0.84	0.01
(3300, 3400)	472.42	4.23	-2.67	0.61	0.82	0.01
(3500, 3600)	490.73	6.44	-3.40	0.93	0.77	0.02
**Axial**						
(2900,3000)	439.16	1.79	-1.89	0.80	0. 0.65	0.09
(3100,3200)	460.38	4.17	-4.13	1.86	0.62	0.11
(3300,3400)	479.43	8.27	-4.63	3.69	0.34	0.30
(3500, 3600)	500.99	15.16	-6.76	6.77	0.25	0.39
**Radial**						
(2900, 3000)	438.12	1.93	-2.87	0.69	0.81	0.01
(3100, 3200)	457.97	2.35	-4.49	0.84	0.88	<0.01
(3300, 3400)	475.21	3.24	-3.40	1.16	0.69	0.04
(3500, 3600)	492.60	7.06	-3.61	2.52	0.34	0.02

## Discussion

Cortical porosity is a key characteristic governing the mechanical and structural behaviour of bone and its quantification is therefore critical for understanding and monitoring the development of various osteopathologies such as osteoporosis [5,9–11,13]. However, non-invasive methods to assess porosity and thereby aid clinical decision-making and medical research remain severely lacking. This study has therefore investigated the ability of low-frequency ax-QA to quantify porosity, and has demonstrated for the first time the sensitivity of phase velocity of acoustic waves to overall porosity as well as individual pore characteristics (pore size, radial pore number, axial pore number). The presented work on porosity is the first step of a structured approach to systematically investigate the influence of individual material properties using laser sintered bone phantoms to develop ax-QA techniques able to measure these individual properties. This work therefore contributes an important milestone towards the systematic development of a comprehensive multi-frequency, multi-mode assessment of human cortical bone for use in clinical applications and bone research [16,23].

The employment of a 2D-FFT approach allowed the analysis of the full wave signal, as opposed to conventional methods that only consider a small part of the wave signal (SOS, FAS, ELA). Additionally, this approach permitted the differentiation between the reflections propagating in the specimen, which is a critical aspect for successfully applying ax-QA in complex systems such as relatively short human bones.

Due to the intrinsic sensitivity of low-frequency wave modes to geometry and material properties, the bone phantoms had to be designed with simple geometry and material properties, while also varying the porosity. Laser sintering using PA12 was shown to be an efficient way to fulfil these requirements, offering a high degree of controllability while showing no influence due to manufacturing direction. While PA12 as a material is only approximately similar to real cortical bone, which has higher elastic moduli and is both heterogeneous and anisotropic, PA12 is very similar to materials that have successfully been used in other phantom studies [23,34,35,38,50] and was therefore deemed appropriate for the presented phantom study.

One critical aspect to consider is that laser sintering leaves residual powder in the pores, and therefore completely vacant pores were not realisable in this study. As a consequence, it is possible that the actual amount of porosity exhibited by the phantoms was lower than planned, indicating that the sensitivity of phase velocity to porosity could be even higher than estimated by our results. Likewise, pores in real cortical bone are non-empty, and thought to be filled with marrow–however, as the acoustic properties of the residual sintering material could not be determined in this experiment, it remains to be investigated whether such partial, non-solid filling can adequately reflect the situation occurring in real bone, with regards to e.g. impedance mismatch.

The phase velocity results also showed a consistent deviation for individual phantoms when compared to the corresponding regression lines across all frequency ranges, which might indicate that the manufactured phantoms had slightly different porosities than specified by the design parameters.

The overall measurement uncertainty remains a surprisingly underreported experimental measure in the literature, with most experiments only reporting the uncertainty for the wave excitation and analysis procedure, thus ignoring the effects of sensor and transducer positioning, as well as pressure between transducer/sensor and phantom. As the current and a previous study [44] have shown, these effects are the dominant contributions to overall measurement uncertainty in both in-vitro and in-vivo measurements—so any estimates that do not take them into consideration will strongly underestimate their true values. In our study, the overall uncertainty in determining the phase velocity was demonstrated to be sufficiently low for applications to potentially differentiate between healthy and osteoporotic bone. However, further improvements to the experimental procedure, such as transducer and sensor placement, could allow for even higher sensitivities, making applications such as monitoring gradual changes in bone properties in individuals possible.

A clear dependence of phase velocity on frequency and porosity produced by increasing pore size or radial pore number was demonstrated, with the velocity decreasing by between 2.5–4.5 m/s per percent of additional porosity (m/(s %p)), with higher frequencies showing stronger changes. This decrease corresponds to -0.5% to -1.0% of wave speed per percent of additional porosity (%v/%p). While the change in phase velocity due to axial pore number was consistent with the results due to pore size and radial pore number, the relative uncertainties for the estimates were too high to draw any conclusions for this parameter. We attribute these high relative uncertainties to the fact that the porosity range covered by changes in axial pore number was only 4% and thus narrower than for the other parameters of porosity. It is therefore entirely plausible that the observed trends also hold true for axial pore number, but this remains to be confirmed in further studies.

Predictions from Timoshenko beam theory agree with the experimentally determined phase velocities, with a consistent 10% underestimation of the predicted absolute values at all frequencies. We attribute this discrepancy to either the residual powder present in the pores resulting from sintering process, or the uncertainty in the material properties of the phantom, which were not nearer specified by the manufacturer, for example introduced through the laser sintering process itself [51]. While the original Timoshenko theory does not explicitly model porosity, porosity was included through a linear effect on the density and a cubic effect on the elastic modulus [52]. Using these approximations, the model predicted a phase velocity change of -1.87 m/(s %p) at 3kHz and -1.98 m/(s %p) at 3.6kHz, agreeing with the range of measured values but predicting a weaker dependency on frequency than observed in our experiment. While this crude approximation yields surprisingly good agreement with the observed data, the inclusion of effects due to pore orientation and distribution might allow the prediction of the mechanical behaviour to be further improved [53–55].

Unexpectedly, the relative changes in wave velocity due to porosity found in this study were comparable to results of experimental and computational studies measuring FAS at high frequencies between 500kHz and 2MHz: An experimental study on 10 human radii found FAS changes of -52 ± 12 m/(s %p), which corresponds to -1.5%v/%p [26], while two computational studies predicted changes between -0.6 and -1.0%v/%p [27,28]. Compared to these studies, limitations of the presented work (-0.5 to -1.5%v/%p) were the simple cylindrical shape, as well as the large size and the regular distribution of pores in the tested phantoms. While the goal of these phantoms was to approximate human cortical bone, it is clear that the direct extrapolation of the presented results to in-vivo measurements in humans may be limited. Through necessity, the systematic bottom-up approach employed in this study restricted the phantom’s geometry to a simple cylinder, which had the additional advantages of being analytically tractable and avoiding potential step-artefacts from the sintering process. Furthermore, although the pore diameter of human cortical bone is known to vary from 50μm to 100–300μm [5,42,56], the achievable pore size in our simplified phantoms was restricted by the limitations of the manufacturing process. However, it is likely that the interaction between pores of smaller diameter d and higher frequencies f will follow the same rules as long as the factor d * f remains constant. While this scaling effect might explain the good agreement between our results and the aforementioned studies, the differences in assessed specimens, geometry, penetration depth, porosity (anisotropy, distribution), and analysis method (FAS, vs full wave), make it likely that the underlying processes are more intricate than suggested by such a simple scaling law.

Another study investigated the effect of cancellous bone porosity on direct transmission velocity (i.e. non-axial) by means of Leeds bone phantoms, which are gelatine granules embedded in an epoxy matrix, with a total porosity of 45–83%. Over this porosity range, the phase velocity at 600 kHz decreased from 2200 m/s to 1600 m/s following a second-order polynomial function, which for comparative purposes corresponds to a linear change of approximately -18 m/(s %p) or -1%v/%p [57]. While the underlying method and employed frequency are considerably different to the studies discussed so far, these results could indicate a non-linear dependency of the phase velocity on porosity at high porosity values.

Since our results indicate an increasing sensitivity to porosity with increasing frequency below a frequency of 10kHz and results from other studies indicate a lower sensitivity at frequencies above 1MHz, it seems probable that the frequency most sensitive to porosity can be found within this frequency range. However, it remains unknown whether specific pore characteristics (size, distribution, connectivity, orientation…) can be assessed independently from one another, and which frequencies and wave modes are most appropriate. Therefore, we suggest that further work should investigate the influence of such pore characteristics using multiple wave modes with a focus on the frequency range between 10kHz and 1MHz. Coupled with investigations extending previous work to assess the influence of locally varying cortical thickness, such work would allow for the assessment of how open-celled porosity at the endosteal surface affects wave propagation.

Together with the presented results, the insights gained from such studies could allow the determination of the ideal wave modes and frequencies to measure clinically relevant porosity and individual pore characteristics.
